# Enhancer/gene relationships: Need for more reliable genome-wide reference sets

**DOI:** 10.3389/fbinf.2023.1092853

**Published:** 2023-02-24

**Authors:** Tristan Hoellinger, Camille Mestre, Hugues Aschard, Wilfried Le Goff, Sylvain Foissac, Thomas Faraut, Sarah Djebali

**Affiliations:** ^1^ IRSD, Université de Toulouse, INSERM, INRAE, ENVT, Univ Toulouse III - Paul Sabatier (UPS), Toulouse, France; ^2^ INSA Toulouse, INP-ENSEEIHT, Toulouse, France; ^3^ GenPhySE, Université de Toulouse, INRAE, INPT, ENVT, Toulouse, France; ^4^ Institut Pasteur, Université Paris Cité, Department of Computational Biology, Paris, France; ^5^ Program in Genetic Epidemiology and Statistical Genetics, Harvard T.H. Chan School of Public Health, Boston, MA, United States; ^6^ Sorbonne Université, INSERM, Institute of Cardiometabolism and Nutrition (ICAN), UMR_S1166, Paris, France

**Keywords:** gene expression regulation, identification of enhancer/gene relationships, method evaluation, chromatin structure, eQTL, functional genomic data, genetic screening

## Abstract

Differences in cells’ functions arise from differential activity of regulatory elements, including enhancers. Enhancers are cis-regulatory elements that cooperate with promoters through transcription factors to activate the expression of one or several genes by getting physically close to them in the 3D space of the nucleus. There is increasing evidence that genetic variants associated with common diseases are enriched in enhancers active in cell types relevant to these diseases. Identifying the enhancers associated with genes and conversely, the sets of genes activated by each enhancer (the so-called enhancer/gene or E/G relationships) across cell types, can help understanding the genetic mechanisms underlying human diseases. There are three broad approaches for the genome-wide identification of E/G relationships in a cell type: 1) genetic link methods or eQTL, 2) functional link methods based on 1D functional data such as open chromatin, histone mark or gene expression and 3) spatial link methods based on 3D data such as HiC. Since 1) and 3) are costly, the current strategy is to develop functional link methods and to use data from 1) and 3) as reference to evaluate them. However, there is still no consensus on the best functional link method to date, and method comparison remain seldom. Here, we compared the relative performances of three recent methods for the identification of enhancer-gene links, TargetFinder, Average-Rank, and the ABC model, using the three latest benchmarks from the field: a reference that combines 3D and eQTL data, called BENGI, and two genetic screening references, called CRiFF and CRiSPRi. Overall, none of the three methods performed best on the three references. CRiFF and CRISPRi reference sets are likely more reliable, but CRiFF is not genome-wide and CRiFF and CRISPRi are mostly available on the K562 cancer cell line. The BENGI reference set is genome-wide but likely contains many false positives. This study therefore calls for new reliable and genome-wide E/G reference data rather than new functional link E/G identification methods.

## 1 Introduction

Vertebrate organisms are made of billions of cells that all have the same genome, but able to deliver a wide range of biological functions. These functional differences are conveyed by the differential expression of genes across cell types, which is partly driven by the differential action of their regulatory elements (promoters, enhancers, insulators, etc.). Among those regulatory elements, enhancers are particularly interesting, not only because they are predominant and cover more genomic space (Pennacchio et al. (2013)), but also because they appear to play important roles in human diseases (Zhang et al. (2018); Nasser et al. (2021)). Enhancers, like promoters, are DNA elements bound by transcription factors (TF). They are known to activate the expression of one or several genes by getting physically close to their promoters in the 3D space of the nucleus (Krivega and Dean (2012); Schoenfelder and Fraser (2019)). There are several publicly available catalogs of enhancers covering many different cell types, especially for the human and the mouse genomes. Enhancers are typically identified experimentally, as, for example, in the VISTA catalog^1^, or bioinformatically, according to functional genomic data: a combination of open chromatin, histone modification and insulator data in the case of the ENCODE catalog (Moore et al. (2020b)), and Cap Analysis Gene Expression (CAGE) data in the case of the FANTOM catalog (Andersson et al. (2014)). Nevertheless, the degree of reliability and the coverage of these catalogs remains limited.

The identification of enhancers and associated genes, i.e., which genes are the targets of which enhancers in a particular cell type, is an important objective in the field. There is increasing evidence that variants associated with common diseases are located in enhancers active in cell types relevant to these diseases (Corradin and Scacheri (2014); Kundaje et al. (2015)). Understanding the enhancer/gene (E/G) relationships active in these particular cell types can help pinpointing important and potentially new genes associated with these diseases, and prioritizing variants in the context of genome-wide association studies (Nasser et al. (2021)). Nonetheless, this task faces important challenges because of the multivariate nature of the enhancer/gene relationship. Indeed, enhancers may 1) be far away from the genes they activate (up to several Mbp), 2) act either upstream or downstream from the activated genes, 3) activate several genes, and 4) need other enhancers to activate a given gene (Krivega and Dean (2012); Schoenfelder and Fraser (2019)).

**FIGURE 1:**
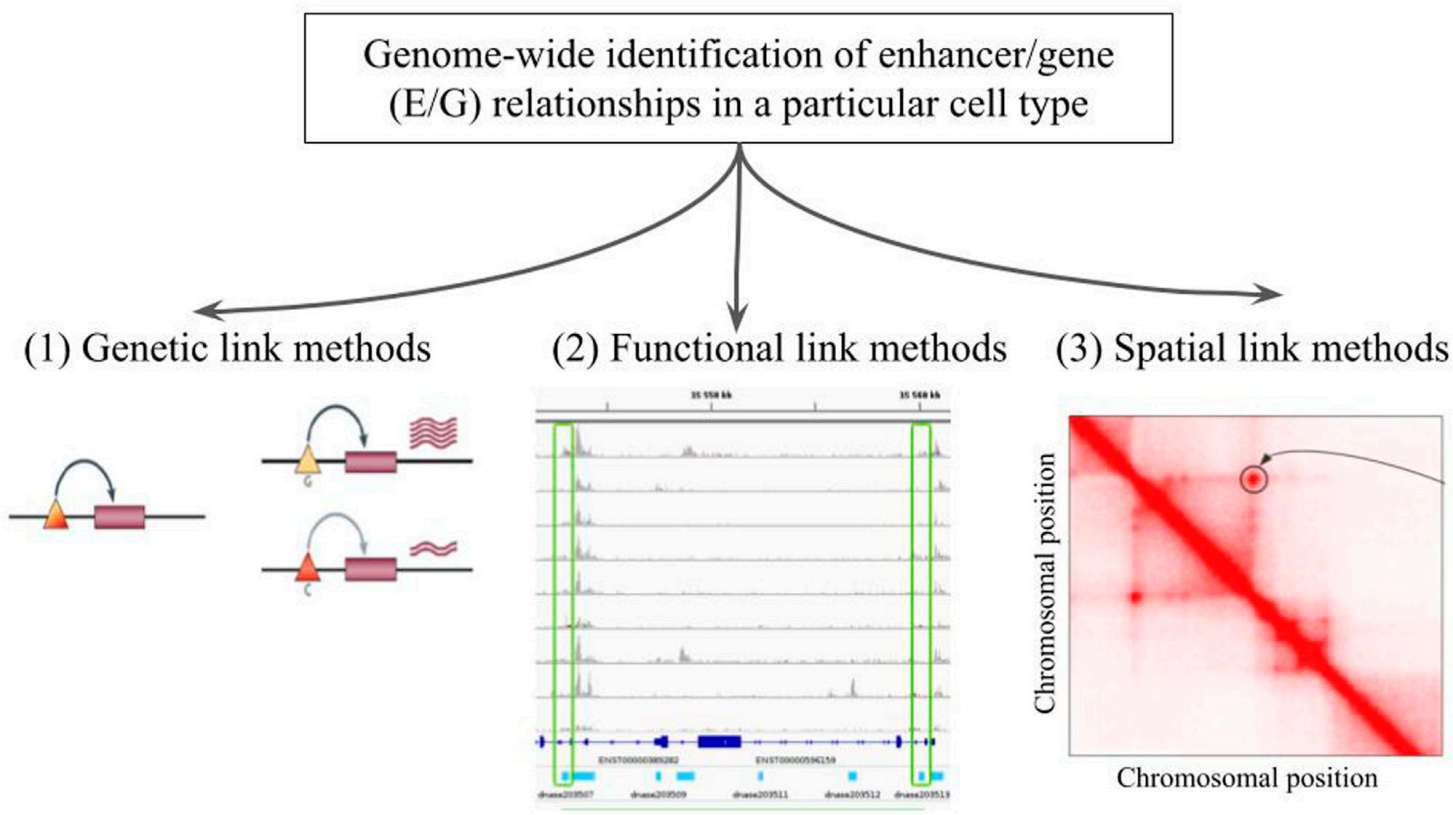
The genome-wide identification of enhancer/gene (E/G) relationships in a particular cell type. Illustration of the three broad approaches that have been described in the literature: **(1)** genetic link methods, **(2)** functional link methods and **(3)** spatial link methods. In panel (1) taken from Cheung and Spielman (2009), the triangles and rectangles represent genetic variants and genes, respectively. When the variant is G the gene is highly expressed, and when it is C the gene is lowly expressed. This variant is said to be an eQTL of the gene, and if located in an enhancer the relationship between the variant and the gene becomes an E/G. Panel (2) illustrates a typical heuristic functional link method, which correlates chromatin accessibility in promoters and enhancers across several cell types and is described in more details in Figure 2 below. Panel (3) represents a squared heatmap where both the horizontal and the vertical axes represent the same portion of the genome divided into equal size bins. The darker the red in the cell, the closer the two regions in the 3D space of the nucleus according to HiC data. Apart from the diagonal, some points far from the diagonal indicate relationships that could be E/G if one of the bin lies in an enhancer and the other one lies at the transcription start site (TSS) or promoter of a gene.

There are three broad approaches that are currently used for the genome-wide identification of E/G relationships in a given cell type (Figure 1): 1) genetic link methods that identify eQTL genetic variants, potentially located in regulatory elements such as enhancers, using expression data (microarray, RNA-seq) applied to a given cell type (Bahcall (2015); Kerimov et al. (2021)), 2) functional link methods that directly identify E/G using genome-wide functional genomic 1D data (open chromatin, histone mark, TF, gene expression) in one or several cell types (see next section), and 3) spatial link (3D) methods that predict E/G using a combination of genome-wide 1D and 3D data (promoter capture HiC, ChiA-PET, etc.) in a given cell type, under the assumption that true E/G relationships are in proximity in 3D space (Jung et al. (2019); Tang et al. (2015)).

Because genetic 1) and spatial link 3) methods are very costly and the generation of 3D data in spatial link methods requires a specific expertise, functional link methods 2) have become the most widely used approach to identify E/G relationships. This is confirmed by the plethora of functional link methods that have been developed since 2011 (see below). On the other hand, data underlying methods of types 1) and 3) are commonly considered as references to assess the reliability of methods of type 2) (see Section 2).

**FIGURE 2:**
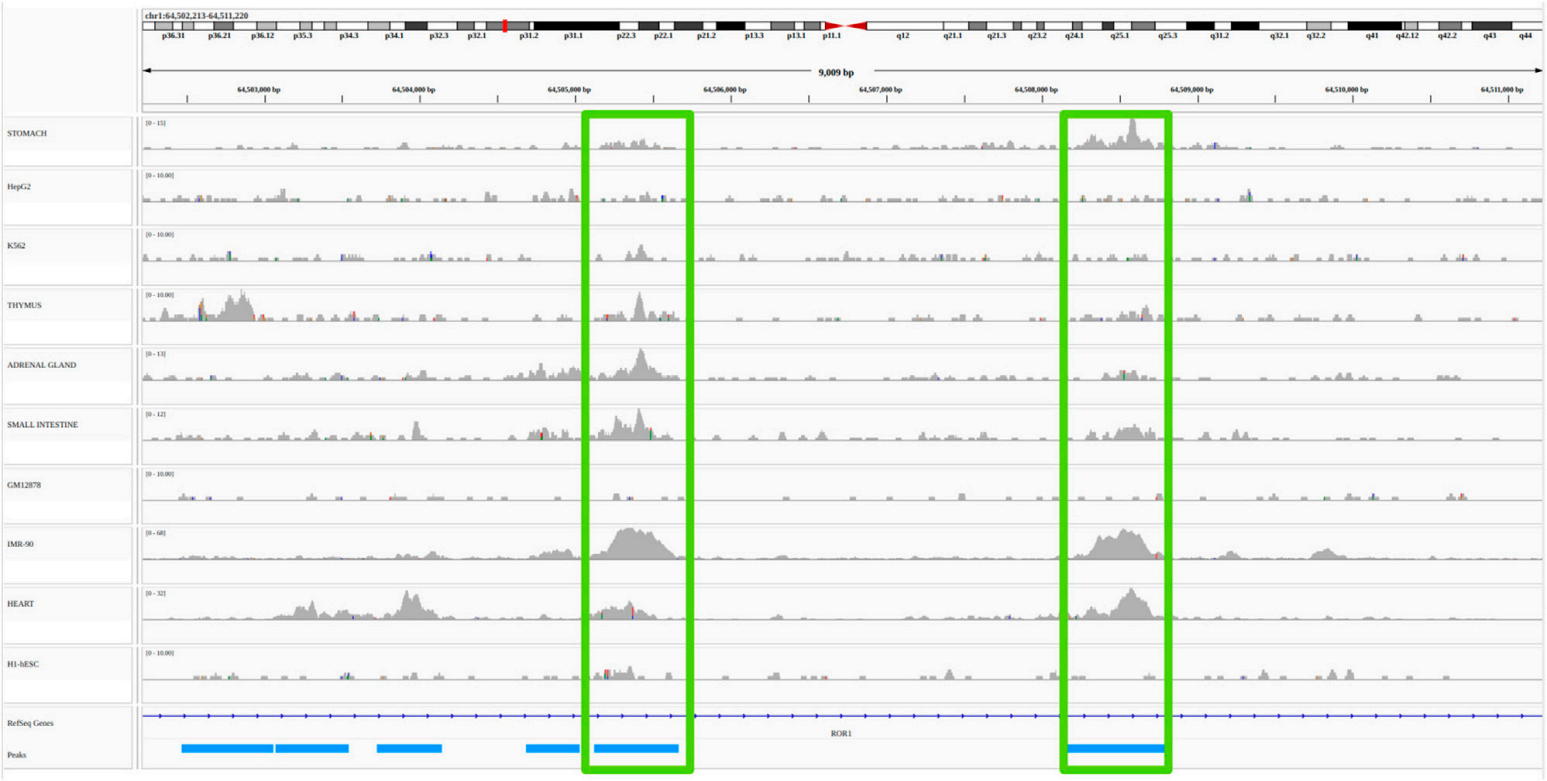
Example of a non-supervised/heuristic method for the identification of E/G in a cell type: the open chromatin correlation method. A common approach to identify candidate E/G is to investigate the correlation between chromatin accessibility signal at two regions across several cell types. The plot represents a portion of the human genome (from position 64,502,213 to position 64,511,220 of chromosome 1 on the hg19 human genome assembly) in IGV (Integrative Genomics Viewer). The horizontal tracks represent ENCODE DNAse-seq signal in 10 different cell types, followed by gene annotation (Refseq) in dark blue and by DNAse-seq consensus peaks from the 10 cell types in light blue. The vertical green rectangles highlight two consensus peaks (and their signal) that have a high (more than 0.7) Pearson correlation between the *log*10 of their normalized accessibilty across the 10 cell types (see *Material and methods*). If one of the consensus peaks was at the transcription start site (TSS) of a gene, then it would typically be interpreted as an E/G.

Functional link methods, also reviewed in Hariprakash and Ferrari (2019), can be divided into two broad categories: non-supervised/heuristic methods, and supervised machine learning methods. While the former generally use few types of functional genomic data in a large number of cell types, the latter use many types of functional genomic data in a reduced number of cell types. Broadly speaking, non-supervised methods use correlations between functional genomic signals present at enhancers and promoters across many cell types. Distance between promoters and enhancers as well as correlation thresholds are determined heuristically and the evaluation of the accuracy of the method is done *a posteriori* using external reference data (most often 3D or genetic) (Ernst et al. (2011); Shen et al. (2012); Thurman et al. (2012); Sheffield et al. (2013); Andersson et al. (2014); Corradin et al. (2014); Yao et al. (2015); Fulco et al. (2019); Moore et al. (2020a)). For illustration purposes, an example of such unsupervised/heuristic methods, the open chromatin correlation method, is provided in Figure 2 (see *Material and Methods* for details).

The second category of methods uses machine learning approaches such as random forests or neural networks. They consist in training a model to discriminate true vs false E/G based on distinctive features from the 1D data they use, from a reference dataset of known E/G (ground positives, most often a combination of 1D data for enhancer and promoter identification and 3D or genetic data for the relationship identification), and a dataset of unsupported E/G (ground negatives), as a negative control. When provided with new data, the model determines which E/G are more likely to be true (Rödelsperger et al. (2011); Aran et al. (2013); He et al. (2014); Roy et al. (2015); Whalen et al. (2016); Cao et al. (2017); Yang et al. (2017); Hait et al. (2018); Li et al. (2019); Belokopytova et al. (2020); Hong et al. (2020); Fan and Peng (2022)).

## 2 Evaluating the most recent functional link methods

Two recent studies evaluated functional link methods (Fulco et al. (2019); Moore et al. (2020a)). However, they did not evaluate the same methods and did not rely on the same reference data. In order to extend the evaluation of existing methods, we assessed the best performing methods of these two studies on the two reference sets they proposed. We also included a third reference set from a recent extended genetic screening analysis (Gasperini et al. (2019)).

The first study (Fulco et al. (2019)) proposes a new unsupervised/heuristic method called the Activity-By-Contact (ABC) model that performs best in their evaluation. The second study (Moore et al. (2020a)) separately evaluates unsupervised/heuristic and supervised machine learning methods. Within the first category, they propose a new method, called Average-Rank, that performs best within its category, while in the second category they identify TargetFinder (Whalen et al. (2016)) as the best performing one. TargetFinder also performed best overall. Those are the methods that will be evaluated here, together with the simplest baseline distance method, that consists in assigning an enhancer to its closest gene.

### 2.1 Description of the evaluated methods

The ABC model defines the score of a potential E/G in a cell type as the product of the activity of the potential enhancer *E* in this cell type, and the contact between *E* and gene *G*, divided by the sum of the same products but across all potential enhancers in a 5 *Mb* region from *G*. The ABC model starts by defining candidate regulatory regions *E*, as regions of open chromatin (defined by either DNAse-seq or ATAC-seq) in a cell type. It then quantifies the enhancer activity (*A*) of these regions *E* by computing the geometric mean of the read counts of chromatin accessibility (usually assessed using DNAse-seq or ATAC-seq) and H3K27ac ChIP-seq at *E*. The contact (*C*) between *E* and *G* is then computed either as the Knight-Ruiz (KR) matrix-balancing normalized Hi-C contact frequency between *E* and the promoter of gene *G*, if cell type specific Hi-C data are available, or simply as the inverse of the distance (fractal globule model) between *E* and *G* otherwise (Fulco et al. (2019)). In order to predict E/G only for expressed genes, the ABC model can either take cell type specific gene expression data in, or consider as a proxy of gene expression, the activity of its promoter as defined above using chromatin accessibility and H3K27ac ChIP-seq data^2^.

The Average-Rank method defines the score of a potential E/G as the inverse of the average of the ranks provided by the Sheffield and the distance methods (Moore et al. (2020a)). The Sheffield method was introduced in 2013 and defines the score of a potential E/G as the Pearson correlation between the logarithm of the chromatin accessibility at *E* (assessed by DNAse-seq) and the logarithm of the expression of *G* across many cell types (Sheffield et al. (2013)). The distance method scores a potential E/G as the inverse of the distance between *E* and *G*. Here potential enhancers are all distal enhancer elements (distal enhancer like signature elements or dELS) of the ENCODE registry of candidate cis-regulatory elements (cCREs) (Moore et al. (2020b)).

TargetFinder defines true (ground positive) E/G based on 3D data (HiC) and learns features associated to those using gradient boosting. The learnt features are as diverse as open chromatin, methylation, histone marks or transcription factors, and can both be taken from enhancer and promoter regions and from the window between them (Whalen et al. (2016)). Indeed its authors showed that features located in enhancer-promoter windows (EPW) are also predictive of true E/G relationships and should be incorporated in the model.

The two first link methods mentioned above also propose their own reference/evaluation datasets.

### 2.2 Description of the reference sets used for the evaluation

Fulco et al. (2019)’s reference set is based on previous CRISPR-based experiments performed in K562 cells and on the output of a new genetic screening technique developed by the authors, called CRISPRi-FlowFISH. This technique was specifically designed to predict E/G in a cell type for a given small number of genes. As stated by the authors, it perturbs “hundreds of non-coding elements in parallel and quantifies their effects on the expression of an RNA of interest, combining CRISPR interference, RNA fluorescence *in situ* hybridization (FISH) and flow cytometry”. In this approach, they “deliver KRAB-dCas9 to many candidate regulatory elements in a population of cells by using a library of guide RNAs”. The results of this technique are then subjected to a statistical framework to determine the sets of E/G that are active and inactive in the cell type. The technique was then applied to thirty genes in five genomic regions (spanning 1.1–4.0 *Mb*) for which they tested all DNase I hypersensitive (DHS) elements (representing open chromatin regions) in K562 cells within 450 *kb* of the gene of interest. Together with previous CRISPR experiments, this approach yielded 109 ground positives (i.e., “positive in the evaluation set”) and 3,754 ground negatives (i.e., “negative in the evaluation set”) E/G, which are considered as a reference set for the evaluation of numerous methods of the field including the ABC model and the distance method (Fulco et al. (2019)). We will use this reference set here and call it CRiFF (Table 1). Note that the 30 selected genes had an RPKM expression level above 20 in K562, and that some of them were erythroid-specific while others were ubiquitous. No filtering on chromatin accessibility level was applied to open chromatin regions, however the sequences of the probes that were designed to target open chromatin regions through gRNAs had to be specific enough. Fulco et al. also garantee a 5% FDR to detect E/G and more than 80% power to detect a 25% effect on gene expression with CRiFF. Finally it has to be noted that the 109 ground positives and the 3,754 ground negatives of CRiFF exclude repressive elements and promoter-promoter interactions (interactions where the targeted element is located less than 500*bp* away from a TSS).

**TABLE 1:** Number of ground positive and ground negative E/G relationships for each of the three evaluation sets considered, namely, BENGI, CRiFF and CRISPRi.

Evaluation set (cell type)	Source data type	# Ground positive E/G Relationships	# Ground negative E/G Relationships
BENGI (GM12878)	GEUVADIS eQTL	2,073	48,926
	CHi-C	88,245	287,483
	CTCF ChIA-PET	7,591	97,425
	GTEx eQTL	1,301	36,899
	HiC	3,404	150,335
	RNA polII ChIA-PET	23,699	133,536
CRiFF (K562)	CRiFF	109	3,754
CRISPRi (K562)	CRISPRi	651	24,576

To complement CRiFF which is rather small, we decided to use another recent genetic screening set that differs from CRiFF in being enhancer-centric instead of gene-centric. It was also available on K562 and could be retrieved from Moore et al. (2020a)’s reference set. It is made of 651 ground positive and 24,576 ground negative E/G relationships, and we will call it CRISPRi (Table 1).

Moore et al.‘s reference set is entitled BENGI (Benchmark of candidate Enhancer-Gene Interactions) and is made of sets of E/G active and inactive in different cell lines according to different types of data (3D, genetic). We focus our evaluation on the GM12878 cell line, which has the largest amount of annotation data, with 6 sets of active and inactive E/G available. The active E/G sets result from the processing of four types of 3D data, Hi-C (Rao et al. (2014)) and promoter capture Hi-C (Mifsud et al. (2015)) data and ChiA-PET of polymerase II and CTCF (Tang et al. (2015)) data, and of eQTL data from two different studies, GEUVADIS (Lappalainen et al. (2013)) and GTEx (Consortium et al. (2015)). The sets of ground negatives are built by taking, for each enhancer of a positive set, all the genes not connected to it in the positive set and lying within the 95 percentile of the positive set distances from it. The number of ground positive and negative E/G obtained are indicated in Table 1. Since 3D and eQTL data are not specifically generated to identify E/G relationships, the BENGI reference sets are expected to be overall less reliable than the CRiFF and the CRISPRi reference sets. However, the fact that BENGI provides genome-wide information is an advantage over CRiFF.

Given all these data we proceeded to the evaluation of the ABC model, the Average-Rank method, the distance method, and TargetFinder, on all three reference datasets: BENGI, CRiFF and CRISPRi.

### 2.3 Description of the evaluation

**FIGURE 3:**
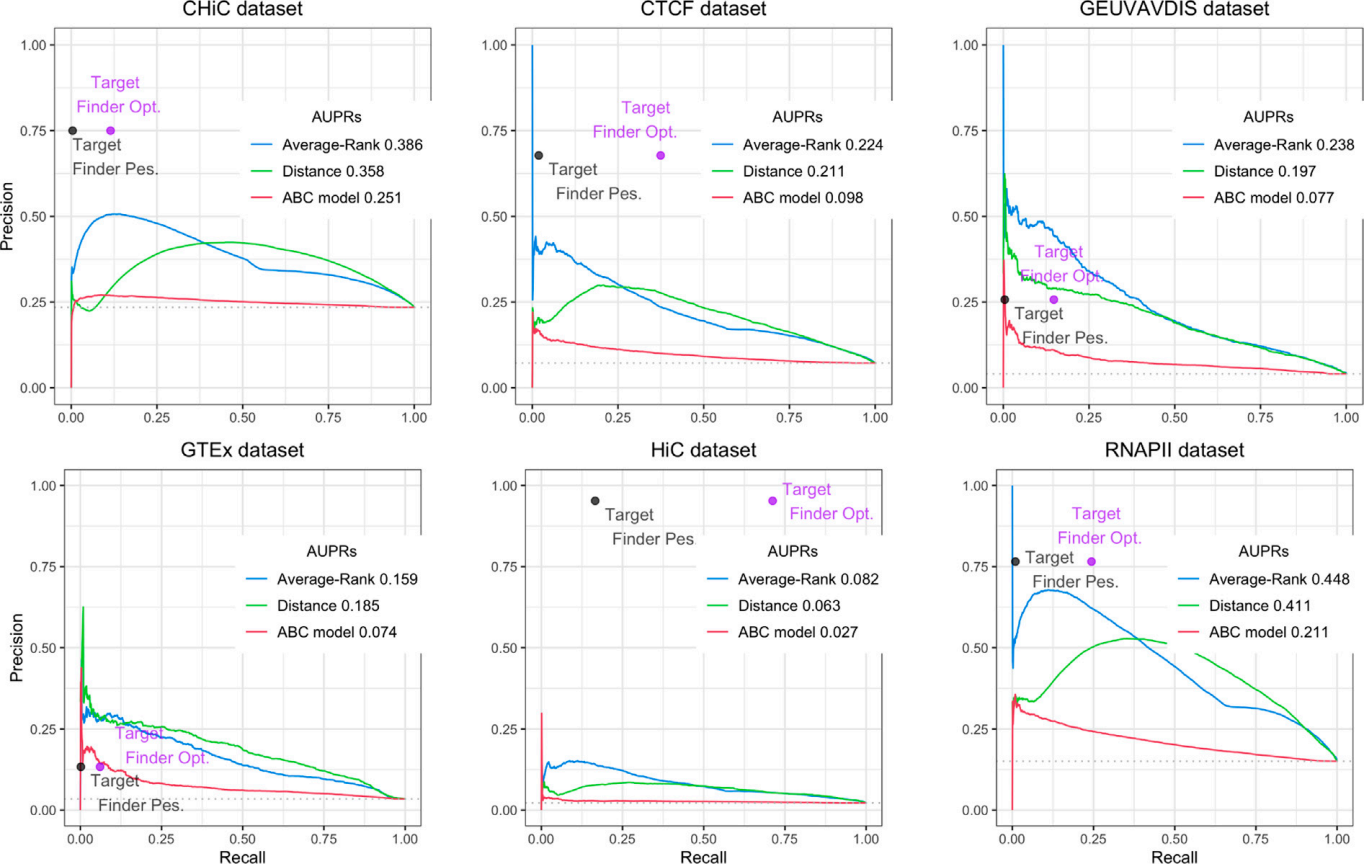
Performances of Average-Rank, distance, ABC model and TargetFinder methods on the six datasets of the GM12878 BENGI evaluation set (all pairs, natural ratio). For each method except TargetFinder, a Precision-Recall curve and an AUPR (Area Under the Precision-Recall curve) are provided. For TargetFinder, the two dots, TargetFinder_pes and TargetFinder_opt, correspond to two different ways of computing recall, a pessimistic and an optimistic one (see *Material and methods* and Table 2 for more details).

**FIGURE 4:**
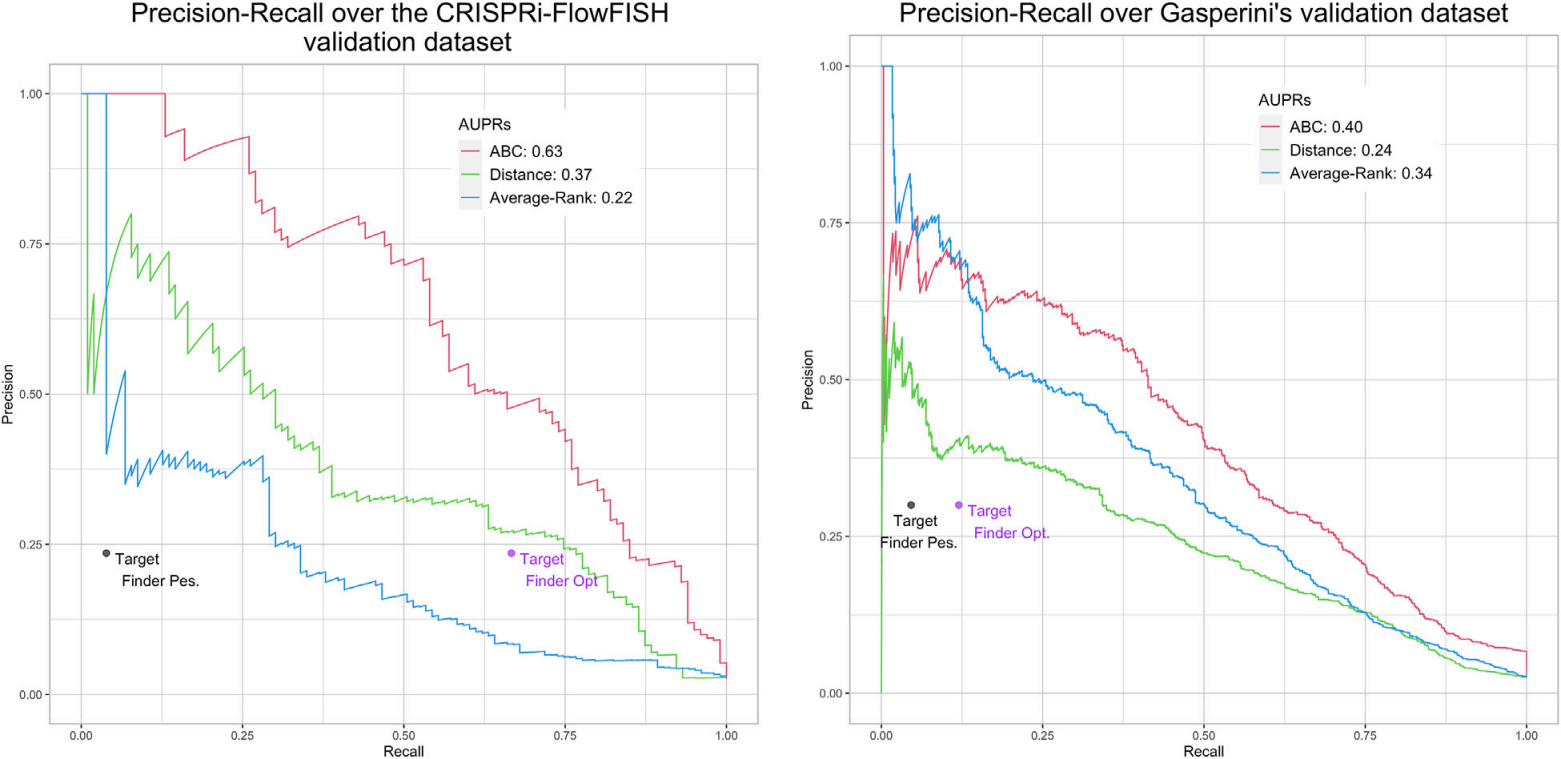
Performances of ABC model, distance, Average-Rank and TargetFinder methods on the K562 CRiFF **(A)** and CRISPRi **(B)** evaluation sets. For each method except TargetFinder, a Precision-Recall curve and an AUPR (Area Under the Precision-Recall curve) are provided. For TargetFinder, the two dots, TargetFinder_pes and TargetFinder_opt, correspond to two different ways of computing recall, a pessimistic and an optimistic one (see *Material and methods* and Table 2 for more details).

For the ABC model, the Average-Rank method and the distance method, we used the code provided by the authors (Fulco et al. (2019); Moore et al. (2020a)), with some adjustments, while for the last one we downloaded the predictions provided by the authors (Whalen et al. (2016))^3^. The obtained results are presented on Figure 3 for BENGI, and on Figure 4 for CRiFF and CRISPRi (see *Material and methods*). Note that while Fulco et al. provided a code associated to their proposed method^4^, Moore et al. only provided a code for the evaluation of their proposed method on a given evaluation set, which is less generic^5^. In addition, the fact that we used TargetFinder’s already thresholded predictions only allowed us to compute a single pair of (precision, recall) values for each reference set, and explains the absence of AUPR curves for this tool. In fact, two different pairs of (precision, recall) values, a pessimistic one and an optimistic one, were computed and plotted for TargetFinder, leading to two different dots for TargetFinder on the plots, TargetFinder_pes and TargetFinder_opt (see *Material and methods* for details).

We replicated the results of the two evaluation papers (Fulco et al. (2019); Moore et al. (2020a)). The curves and AUPRs (Area Under the Precision-Recall curve) of the Average-Rank and the distance methods of Figure 3 are in agreement with Figure S2 of Moore et al. (2020a) derived for GM12878 cell line (all pairs, natural ratio). Similarly, the curves and AUPRs of the ABC model and the distance method of Figure 4A agree with Figure 3A of Fulco et al. (2019). Like Moore et al. (2020a), we also found that TargetFinder performs better than Average-Rank, except on eQTL reference sets. Altogether, these positive controls confirmed the validity of the pipeline we implemented.

Figure 3 further shows low AUPR values for the first three methods on the six BENGI datasets, and that TargetFinder performs best overall, followed by Average-Rank, distance and ABC model. Note that TargetFinder is much more precise than sensitive, and performs much better on HiC and CTCF sets. This last result can be explained by the fact that TargetFinder learns true E/G based on HiC data. Nevertheless, Figure 4A shows larger AUPRs for the three last methods on the CRiFF set, and that the ABC model performs best (*AUPR* = 0.63), before the distance method and finally the Average-Rank method. Contrary to its result on BENGI, TargetFinder does not perform well on CRiFF.

When comparing the performances of the methods on CRISPRi with respect to CRiFF, if the methods that perform best (ABC model) and worst (TargetFinder) are the same, it has to be noted that Average-Rank performs better than distance and that the AUPRs are globally lower (Figure 4B). It is also important to note that on half of the evaluation sets used here, the number of TargetFinder’s validated E/G relationships is lower or equal to 9 (Table 2), showing that this tool is not easily applicable and might only deliver predictions for small subsets of references.

**TABLE 2:** TargetFinder’s performances on BENGI, CRiFF and CRISPRi. *#Predicted* refers to the number of positive (TP + FP) and negative (TN + FN) predictions that were also in the reference set, and is used to compute Precision. *Ground positives pes.* refers to the total number of ground positive elements in the reference set, while *Ground Positives opt.* refers to the subset of those that were also in the set of E/G relationships TargetFinder started from. *Recall pes.* and *Recall opt.* are computed from *Ground Positives pes.* and *Ground Positives opt.* respectively, therefore corresponding to pessimistic and optimistic estimations of recall respectively.

Evaluation set	Source	True positives	#Predicted	Ground positives pes	Ground positives opt	Precision (in %)	Recall pes (in %)	Recall opt (in %)
BENGI (GM12878)	GEUVADIS eQTL	9	35	2,073	61	25.7	0.43	14.75
	CHi-C	342	456	88,245	2,986	75.0	0.39	11.45
	CTCF ChIA-PET	143	211	7,591	382	67.8	1.88	37.43
	GTEx eQTL	2	15	1,301	33	13.3	0.15	6.06
	HiC	564	592	3,404	792	95.3	16.57	71.21
	Pol II ChIA-PET	222	290	23,699	911	76.6	0.94	24.37
CRiFF (K562)	CRiFF	4	17	103	6	23.5	3.88	66.67
CRISPRi (K562)	CRISPRi	3	10	651	35	30.0	0.46	12.0

Therefore, state-of-the-art E/G identification methods do not perform very well overall, and using 3D or genetic screening data as reference provides completely opposite answers to the question of the best performing E/G identification method.

## 3 Discussion

The poor performance (small precision values even for small recall values) of the ABC model on the BENGI sets could be due to the fact that BENGI’s underlying data (HiC, promoter capture HiC, ChiA-PET and RNA-seq) were not specifically designed to identify E/G relationships. For instance, some E/G relationships may not need spatial proximity or the presence of CTCF to operate (Ray-Jones and Spivakov (2021)). Likewise the presence of an eQTL in a predicted enhancer does not necessarily imply the presence of an E/G relationship. The poor performances of the Average-Rank and the TargetFinder methods (small precision values even for small recall values) on the CRiFF and CRISPRi data are more difficult to explain as these techniques should be quite exhaustive in identifying the enhancers of a given gene. However, the authors of CRiFF state in their paper that “CRISPRi might fail to discover certain regulatory elements, for example, due to differential sensitivity to KRAB-mediated inhibition” (Fulco et al. (2019)). The CRISPRi set also only includes intergenic enhancers, which could seem quite restrictive knowing that there should be a large number of intronic enhancers as well. Why this would affect the Average-Rank and TargetFinder methods more than the ABC model still requires further investigation. Looking for a good compromise between the two types of evaluations, at first glance, the baseline distance method could appear as the best one, with the most stable results across evaluation sets. However, in addition to the fact that it is one of the worst methods on CRISPRi (Figure 4B), we know this method does not work well in many cases (Krivega and Dean (2012); Mumbach et al. (2017); Nasser et al. (2021)). Altogether our results illustrate the challenge in defining the best approach for E/G inference.

Because they were specifically designed to identify E/G relationships for a selected set of genes or enhancers, the CRiFF and the CRISPRi techniques seem to be better suited to generate true E/G reference/evaluation data. Therefore if we really had to select an E/G relationship identification method, then we would choose the one that performs best on the CRiFF and CRISPRi data, namely, the ABC model. Another, more practical, reason to select the ABC model over the Average-Rank and TargetFinder methods is that a dedicated and more importantly well documented software has been made available to the community by its authors^6^. Implementing the Average-Rank and TargetFinder methods can be more challenging. Finally, contrary to the Average-Rank method that requires gene expression and chromatin accessibility data on many cell types (here 112), and to TargetFinder that requires tens of data types on the cell type of interest, the ABC model only requires two types of data (open chromatin and H3K27ac) on the cell type of interest. This substantially broadens the scope of application, as the actual amount of data available is likely going to be limited in many real data settings.

One of the limitations of the CRiFF technique is that it does not provide genome-wide results. In the present study, the CRiFF data we used cover 58 different genes located in 21 different genomic regions ranging from 1*Mb* to 4 *Mb* in size, which represents less than 1 percent of the genome. The CRISPRi technique is expected to be more representative of the genome, but it produced results that were similar to CRiFF. Another potential bias could come from the use of the K562 cancer cell line which is the only cell line for which there was sufficient CRiFF data. Even if the authors have performed more CRISPRi-FlowFISH experiments since our study (283 true validated and 5,756 false E/G in 11 cell types, Nasser et al. (2021)), this type of reference data remains not genome-wide and biased toward cancer cell lines, like CRISPRi.

Altogether our results call for the generation of more complete and reliable E/G relationship reference/evaluation data, rather than for new more elaborate E/G relationship identification methods, such as the ones that are currently being developed. A more reliable genome-wide set of E/G would indeed allow to better evaluate the numerous already existing E/G relationship identification methods that are based on 1D data (i.e., functional link methods), in order to finally reach a consensus in this field, and be able to answer numerous questions related to cell function and disease.

## 4 Materials and methods

### 4.1 Pairwise chromatin accessibility correlation across cell types

In order to illustrate unsupervised/heuristic enhancer/gene identification methods, we chose the simplest one, the pairwise chromatin accessibility correlation across cell types, and represented it on Figure 2. The actions that led to this figure are the following: we first downloaded the ENCODE uniformly processed read alignments (bam files) of DNAse-seq data (single end) from 10 cell types: stomach, HepG2, K562, thymus, adrenal gland, small intestine, GM12878, IMR-90, heart and H1-hESC, with accession numbers provided in Table 3. We then called the chromatin accessibility peaks from the mapped reads in each cell type using macs2^7^ (Figure 2; Table 3).

**TABLE 3:** ENCODE cell types and accession numbers of associated DNA-seq alignment bam files.

ENCODE cell type	bam file accession number
stomach	ENCFF703DYP
HepG2	ENCFF343CEI
K562	ENCFF224FMI
thymus	ENCFF067LVL
adrenal gland	ENCFF900LLD
small intestine	ENCFF315TUQ
GM12878	ENCFF246VVI
IMR-90	ENCFF775ZJX
heart	ENCFF923SKV
H1-hESC	ENCFF869SQU

We obtained from about 60,000 (GM12878) to about 200,000 (IMR-90) peaks per cell type. By concatenating, sorting and merging on the genome the peaks called in each cell type using bedtools merge, we then obtained 473,766 consensus peaks across all cell types. We then quantified the chromatin accessibility of the 473,766 consensus peaks in each cell type by simply counting the number of mapped reads of each cell type overlapping each consensus peak using bedtools intersect, and normalized the number of reads of each peak in each cell type by the total number of mapped reads in peaks for this cell type. Finally we computed the consensus peak pairwise Pearson correlation between the *log*10 of the normalized chromatin accessibility across the 10 cell types of these peaks for all pairs of peaks less distant than 500 *kb* using a script that we wrote: compute_correlations.py ^8^. We then only considered as E/G relationships, the pairs of peaks with a correlation above 0.7 and for which one of the two peaks overlapped the most 5’ bp (TSS) of a Gencode v19^9^ gene (vertical green rectangles on Figure 2).

### 4.2 Method evaluation

In addition to evaluating the ABC model on BENGI and the Average-Rank and the TargetFinder methods on CRiFF, and since Moore et al. provided the code of the Average-Rank method, and Fulco et al. the code to run the ABC model, we decided to try and reproduce the evaluation of the Average-Rank method on BENGI and of the ABC model on CRiFF. We also used the code of the distance method provided by Moore et al. (with some modifications), to evaluate the baseline distance method on BENGI, CRiFF and CRISPRi (Tables 1, 2; Figures 3, 4).

In total we evaluated four methods, the ABC model, the Average-Rank, the distance and the TargetFinder methods on three references sets, BENGI, CRiFF and CRISPRi (Figures 3, 4). It has to be noted that contrary to the other methods, TargetFinder’s predictions were downloaded directly from its authors’s website^10^, therefore only allowing us to compute a single pair of (precision, recall) values, and not AUPR curves. In fact, we used two different ways to compute TargetFinder’s recall, an optimistic and a pessimistic one, which led to two different dots for this tool in the evaluation plots (see sections below about TargetFinder). In addition, since the code to generate the Precision-Recall curves and the AUPRs was not provided in the papers, we generated our own R code to make these plots using existing R packages. The code used to perform all these analyses was stored in Jupyter notebooks that we provide below, together with additional details about these analyses.

#### 4.2.1 Method evaluation on BENGI

The Moore et al.’s code, reference data and annotation were first downloaded from the BENGI github repository^11^. More precisely the Scripts directory included, on the one hand the scripts to make the BENGI sets, and on the other hand the scripts to run the evaluation of the methods on a given BENGI set (note that other cell types than GM12878 were provided). It is important to bear in mind that the script corresponding to a method was not a generic script allowing to retrieve all the E/G relationships called by this method in a particular cell type, but rather only produces evaluation data of this method on a given BENGI set, i.e., attaches to each true and false E/G of a BENGI set, the score of the method’s associated prediction (to be used to draw the Precision-Recall curves and compute the AUPRs). Since we could not run any of the scripts from Moore et al. without modifying them, sometimes quite deeply, we suspect these scripts were provided to give a general idea of the underlying analyses rather than to be used as such. No mention of program versions were provided neither, which again hampers reproducibility.

##### 4.2.1.1 Evaluating the distance method on BENGI

To evaluate the distance method on the BENGI sets, we used a slightly modified version of the Run-Distance-Method.sh script provided by Moore et al. This script takes as input a string defining the BENGI set (celltype.settype, for instance GM12878.CHiC), the version of the BENGI set (here v3), the mode (here normal), the expression threshold (here 0.2 but this parameter is not used in normal mode) and the output path. In normal mode, this script calls the rank.distance.py script on the set of human TSSs, the set of all cCREs (candidate cis-regulatory elements) and the BENGI set. It then outputs a 2 column file including for each E/G of the BENGI set on a row, 1 or 0 according to whether this E/G is true or false according to the BENGI set and the score provided by the distance method which is defined as the inverse of the smallest distance between a TSS of G and the enhancer E. Our modification consisted in adding two additional columns to this tabulated file, one for the enhancer id and one for the gene id, this for an easier downstream fusion with the evaluation result of the Sheffield method. For this purpose we also had to modify the Run-Distance-Method.sh script so that it sorts the 4 column tabulated file provided by the python script according to the enhancer id and the gene id. After running the evaluation script we plotted the Precision-Recall curves using existing R packages. The following Jupyter notebook provides all the necessary information for evaluating the distance method on the BENGI sets^12^.

##### 4.2.1.2 Evaluating the Average-Rank method on BENGI

To evaluate the Average-Rank method on the BENGI sets, we first had to run the Sheffield method (correlation between open chromatin at E and expression level at G) on each BENGI set.

For this we first downloaded the DNAse Hypersensitivity (DHS) peaks with their chromatin accessibility in 112 cell types (dhs112_v3.bed file) and the genes with their expression levels in the same 112 cell types (exp112.bed file) from the web^13^ and as indicated in page 14 of Moore et al. (2020a). We then ran the Run-Sheffield.sh script that evaluates the Sheffield method on a given BENGI set. This script takes as input a string defining the BENGI set, the version of the BENGI set and the output path. It then makes the set of enhancers of the BENGI set in bed format, the enhancer matrix with these enhancers in rows and their chromatin accessibility in the 112 cell types in columns, the genes of the BENGI set in bed format, the matrix of these genes in rows with their expression levels in the 112 cell types in columns, and then calls the sheffield.correlation.py script. This script takes as input a matrix of gene expression in the 112 cell types, the gene file in bed format, the enhancer matrix, a gene summary file, the BENGI set and the cell type. It then outputs a 6 column file including for each E/G of the BENGI set on a row, 1 or 0 according to whether this E/G is true or false in the BENGI set, the Pearson correlation between the chromatin accessibility at E and the expression level at G across the 112 cell types, the *p*-value, the Z-score, the enhancer id and the gene id.

In fact we had to modify the Run-Sheffield.sh script and the sheffield.correlation.py script to make them work. The complete process to run the Sheffield method on the BENGI sets can be found on this page^14^.

Finally we ran the Run-Average-Rank.sh script that evaluates the Average-Rank method on a BENGI set. This script takes as input the BENGI set and its version, and outputs a 7 column tabulated file including for each E/G of the BENGI set, 1 or 0 according to whether this E/G is true or false in BENGI, the average rank score, the distance score, the correlation score, the distance rank, the correlation rank and the average rank between the distance and the correlation. Here we also had to modify the bash script to make it run but more importantly to correct a bug. The exact process and modifications are provided in^15^.

Once again we plotted the Precision-Recall curves using an R code of our own. The complete process to evaluate the Average-Rank method on the BENGI sets can be found here^16^.

##### 4.2.1.3 Evaluating the ABC model on BENGI

In order to evaluate the ABC model on the BENGI sets, we downloaded the ABC model code from its github repository^17^. Although the complete process is not a pipeline but is rather made of several steps to run one after the other, the documentation was so pedagogic and complete that we had no particular issue running the ABC model on GM12878 data. We also found the tools and associated version to use. Non-etheless and for the sake of reproducibility the complete process is detailed in this notebook^18^.

##### 4.2.1.4 Evaluating TargetFinder on BENGI

In order to evaluate TargetFinder on the BENGI sets, we first downloaded TargetFinder’s GM12878 predictions from the dedicated github repository^19^. We used the GBM classifier including Enhancer-Promoter windows (EPW). The prediction file was made of all true and false GM12878 HiC loops (44,313 in total, of which 2,113 are true and 42,200 are false) associated to whether TargetFinder predicted an E/G or not.

In order to compute TargetFinder’s precision and recall on each of the 6 BENGI sets, we first computed TargetFinder’s true positives (TPs) on each set, i.e., TargetFinder’s predictions that corresponded to a ground positive E/G of the BENGI set. To do so, we first had to convert the enhancer and promoter coordinates of the TargetFinder’s prediction file into cCRE-ELS (candidate cis-regulatory elements with enhancer like signature) ids and gene ids respectively. For that we used bedtools intersect on the GM12878 cCRE-ELS file and the Gencode v19 TSS file from Moore et al. respectively. In total we found 342, 222, 143, 564, 9 and 2 TPs for CHIC, RNAPII ChIA-PET, CTCF ChIA-PET, HiC, Geuvadis and GTEx (Table 2).

Precision was then computed by dividing these numbers by the sum of these numbers and TargetFinder’s false positive predictions according to BENGI.

Recall was computed in two different ways: by dividing the TPs 1) by the total number of BENGI ground positive E/G (Recall_pes, like pessimistic Recall, giving rise to the TargetFinder_pes dot on the plot) and 2) by the subset of BENGI ground positive E/G that were also in the initial set of 44,313 E/G relationships given as input to TargetFinder (Recall_opt, like optimistic recall, giving rise to the TargetFinder_opt dot on the plot).

The performances of TargetFinder on BENGI are indicated on Table 2.

#### 4.2.2 Method evaluation on CRISPRi-FlowFISH (CRiFF)

To obtain the CRiFF reference set we first downloaded Table S6a from Fulco et al. (2019) as a tsv file, and then obtained the 109 ground positive and the 3754 ground negative E/G relationships by performing the filters detailed in^20^ (the ground negatives are defined as either not significant or not associated to a decrease in gene expression).

In order to be able to use almost the same scripts as above for the distance and the Average-Rank methods, we first intersected the enhancers of the CRiFF set with the ENCODE cCRE-ELS (candidate cis-regulatory element with enhancer like signature) provided and used by Moore et al. This process is described in the three notebooks below. We have to say that we only slightly modified the distance and Average-Rank methods scripts used above for BENGI and GM12878 in order to run then on CRiFF and K562 (see notebooks below).

##### 4.2.2.1 Evaluating the distance method on CRiFF

The complete process for this evaluation is provided in the following notebook^21^.

##### 4.2.2.2 Evaluating the Average-Rank method on CRiFF

The complete process for this evaluation is provided in the following notebook^22^.

##### 4.2.2.3 Evaluating the ABC model on CRiFF

The complete process for this evaluation is provided in the following notebook^23^.

##### 4.2.2.4 Evaluating TargetFinder on CRiFF

In order to evaluate TargetFinder on the CRiFF set, we first downloaded TargetFinder’s K562 predictions from the dedicated github repository^24^. We used the GBM classifier including Enhancer-Promoter windows (EPW). The prediction file was made of all true and false K562 HiC loops (41,477 in total, of which 1977 are true and 39,500 are false) associated to whether TargetFinder predicted an E/G or not.

In order to compute TargetFinder’s precision and recall on the CRiFF set, we first computed TargetFinder’s true positives (TPs), i.e., TargetFinder’s predictions that corresponded to a ground positive E/G of the CRiFF set. To do so, we first had to convert the enhancer and promoter coordinates of the TargetFinder’s prediction file into cCRE-ELS (candidate cis-regulatory elements with enhancer-like signature) ids and gene ids respectively. For that we used bedtools intersect on the K562 cCRE-ELS file and the Gencode v19 TSS file from Moore et al. respectively. In total we only found 4 TPs (Table 2).

Precision was then computed by dividing these numbers by the sum of these numbers and TargetFinder’s false positive predictions according to CRiFF.

Recall was computed in two different ways: by dividing the TPs 1) by the total number of CRiFF ground positive E/G (Recall_pes, in reference to pessimistic recall, giving rise to the TargetFinder_pes dot on the plot) and 2) by the subset of CRiFF ground positive E/G that were also in the initial set of 41,477 E/G relationships given as input to TargetFinder (Recall_opt, in reference to optimistic recall, giving rise to the TargetFinder_opt dot on the plot).

The performances of TargetFinder on CRiFF are indicated on Table 2.

#### 4.2.3 Method evaluation on CRISPRi

The K562 CRISPRi set was obtained from the BENGI’s github repository^25^. It included 651 ground positive and 24,576 ground negative E/G (Table 1).

##### 4.2.3.1 Evaluating the distance method on CRISPRi

The evaluation of the distance method on the K562 CRISPRi set was done exactly the same way as on the GM12878 BENGI sets, but replacing the GM12878 cCREs by the K562 cCREs (see above).

##### 4.2.3.2 Evaluating the Average-Rank method on CRISPRi

The evaluation of the Average-Rank method on the K562 CRISPRi set was done exactly the same was as on the GM12878 BENGI sets, but replacing the GM12878 cCREs by the K562 cCREs (see above).

##### 4.2.3.3 Evaluating the ABC model on CRISPRi

To evaluate the ABC model on the K562 CRISPRi set, we had to rerun the ABC model on K562 but using a different white list as the one used for the evaluation on CRiFF. Indeed, the ABC model’s step 1.3 called make candidate region can take as input a white list of promoters and enhancers on which to enforce predictions, and it was important to use it to ensure that all CRISPRi ground positives and negatives could be predicted by the ABC model. Here the white list we used was made of the union of all K562 cCRE-ELS from Moore et al., and of the Gencode v19 TSS from Moore et al. that we extended by 250*bp* on each side.

##### 4.2.3.4 Evaluating TargetFinder on CRISPRi

The evaluation of TargetFinder on the K562 CRISPRi set was done exactly the same way as on the GM12878 BENGI set (see above). The number of TPs was only 3. The performances of TargetFinder on CRISPRi are indicated on Table 2.

## Footnotes

1
http://enhancer.lbl.gov/

2
https://github.com/broadinstitute/ABC-Enhancer-Gene-Prediction

3
https://github.com/shwhalen/targetfinder/

4
https://github.com/broadinstitute/ABC-Enhancer-Gene-Prediction

5
https://github.com/weng-lab/BENGI

6
https://github.com/broadinstitute/ABC-Enhancer-Gene-Prediction

7
https://hoellin.github.io/eg/guidebooks/compute_correlations.html

8
https://github.com/sdjebali/EnhancerGene

9
https://www.gencodegenes.org/

10
https://github.com/shwhalen/targetfinder

11
https://github.com/weng-lab/BENGI

12
https://hoellin.github.io/eg/notes_BENGI/distance_method/distance_evaluation_with_code.html

13
http://big.databio.org/papers/RED/supplement/

14
https://hoellin.github.io/eg/notes_BENGI/dnase_expression_correlation/correlation_method_with_code.html

15
https://hoellin.github.io/eg/notes_BENGI/avg_rank_method/avg_rank_method_with_code.html\#partial-reimplementation-of-run-average-ranksh

16
https://hoellin.github.io/eg/notes_BENGI/avg_rank_method/avg_rank_method_with_code.html

17
https://github.com/broadinstitute/ABC-Enhancer-Gene-Prediction

18
https://hoellin.github.io/eg/notes_ABC/BENGI/notebook_ABC_over_BENGI_GM12878_from_ccRE_ELSs.html

19
https://github.com/shwhalen/targetfinder/blob/master/paper/targetfinder/GM12878/output-epw/predictions-gbm.csv

20
https://hoellin.github.io/eg/notes_ABC/K562/ABC_K562_CRISPRi_FlowFISH.html

21
https://hoellin.github.io/eg/notes_BENGI/CRISPRi_FlowFISH/distance_method/distance_over_fulco_et_al_crispri.html

22
https://hoellin.github.io/eg/notes_BENGI/CRISPRi_FlowFISH/avg_rank_method/avg_rank_method_with_code.html

23
https://hoellin.github.io/eg/notes_ABC/K562/april_K562_56_genes/april_K562_56_genes.html

24
https://github.com/shwhalen/targetfinder/blob/master/paper/targetfinder/K562/output-epw/predictions-gbm.csv

25
https://github.com/weng-lab/BENGI/tree/master/Benchmark/All-Pairs.Natural-Ratio
